# The T-box transcription factor 3 is a promising biomarker and a key regulator of the oncogenic phenotype of a diverse range of sarcoma subtypes

**DOI:** 10.1038/oncsis.2016.11

**Published:** 2016-02-22

**Authors:** T Willmer, A Cooper, D Sims, D Govender, S Prince

**Affiliations:** 1 Division of Cell Biology, Department of Human Biology, Faculty of Health Sciences, University of Cape Town, Cape Town, South Africa; 2 Division of Anatomical Pathology, Department of Pathology, Faculty of Health Sciences, University of Cape Town, Cape Town, South Africa

## Abstract

Sarcomas represent a complex group of malignant neoplasms of mesenchymal origin and their heterogeneity poses a serious diagnostic and therapeutic challenge. There is therefore a need to elucidate the molecular mechanisms underpinning the pathogenesis of the more than 70 distinguishable sarcoma subtypes. The transcription factor TBX3, a critical developmental regulator, is overexpressed in several cancers of epithelial origin where it contributes to tumorigenesis by different molecular mechanisms. However, the status and role of TBX3 in sarcomas have not been reported. Here we show that a diverse subset of soft tissue and bone sarcoma cell lines and patient-derived sarcoma tissues express high levels of TBX3. We further explore the significance of this overexpression using a small interferring RNA approach and demonstrate that TBX3 promotes the migratory ability of chondrosarcoma, rhabdomyosarcoma and liposarcoma cells but inhibits fibrosarcoma cell migration. This suggested that TBX3 may play a key role in the development of different sarcoma subtypes by functioning as either an oncoprotein or as a brake to prevent tumour progression. To further explore this, TBX3 knockdown and overexpression cell culture models were established using chondrosarcoma and fibrosarcoma cells as representatives of each scenario, and the resulting cells were characterized with regard to key features of tumorigenesis. Results from *in vitro* and *in vivo* assays reveal that, while TBX3 promotes substrate-dependent and -independent cell proliferation, migration and tumour formation in chondrosarcoma cells, it discourages fibrosarcoma formation. Our findings provide novel evidence linking TBX3 to cancers of mesenchymal origin. Furthermore, we show that TBX3 may be a biomarker for the diagnosis of histologically dynamic sarcoma subtypes and that it impacts directly on their oncogenic phenotype. Indeed, we reveal that TBX3 may exhibit oncogene or tumour suppressor activity in sarcomas, which suggests that its role in cancer progression may rely on cellular context.

## Introduction

Sarcomas are cancers derived from mesenchymal tissue and while they only account for a small percentage of neoplasms, they represent some of the most aggressive cancers in children, adolescents and young adults.^1, 2^ They therefore contribute to a considerable loss of years of life in comparison with other cancers. Sarcomas are frequently resistant to conventional radiation- and chemo-therapies and the heterogeneity that they exhibit, even within histological subtypes, complicates patient care and limits the options of current therapies.^3^ In light of this, there is a growing appreciation of the need to understand the molecular mechanisms underlying the pathogenesis of individual sarcoma subtypes with the view to identifying more effective diagnostic markers and novel treatment strategies. Indeed, the development of subtype or pathway-specific therapies is a rapidly evolving field and recent advances in understanding sarcoma biology have led to the identification of several molecular determinants of different soft tissue and bone sarcoma subtypes. For example, the identification of c-Kit and PDGFRα mutations in gastrointestinal stromal tumours has led to the successful treatment of these cancers by the tyrosine kinase inhibitor, imatinib.^4^ More recently, monoclonal antibodies targeting insulin-like growth factor type 1 receptor have shown promise in phase I and II clinical trials for the treatment of paediatric sarcomas including osteosarcoma, Ewing sarcoma and rhabdomyosarcoma.^5, 6^ Sorafenib and pazopanib, small-molecule inhibitors of vascular endothelial growth factor receptor, have also shown anticancer activity in leiomyosarcomas, angiosarcomas and synovial sarcomas.^7, 8^ In addition, the mechanistic target of rapamycin inhibitor, AP23573, has shown promising clinical efficacy in patients with advanced soft tissue sarcomas.^9, 10^ It is therefore evident that improved sarcoma cure rates will likely be driven by new types of treatment that target specific deregulated proteins within these tumours.

TBX3 is a T-box transcription factor that plays critical roles in embryonic development but it has also been implicated in a wide range of carcinomas.^11^ For example, it is overexpressed in, among others, a subset of breast carcinomas, melanoma, ovarian, pancreatic, cervical, liver and bladder carcinomas and there is evidence that it contributes to multiple aspects of the oncogenic process.^11^ TBX3 negatively regulates apoptosis in rat bladder^12^ and liver carcinoma,^13, 14^ can bypass senescence and promote proliferation by repressing the key cell cycle regulators p14/p19^ARF^, p21^WAFI/CIPI/SDII^ (referred to as p21) and the tumour suppressor phosphatase and tensin homologue (PTEN).^14, 15, 16, 17, 18, 19^ Importantly, TBX3 plays a critical role in promoting breast tumour and melanoma formation, invasion and metastasis in part through its ability to directly repress the cell adhesion protein E-cadherin.^15, 20, 21, 22, 23, 24^ Although there is compelling evidence to support a direct link for TBX3 in the development of carcinomas, and indeed it has been identified as a novel anticancer drug target, whether it is overexpressed in sarcomas and whether it contributes to oncogenesis in these cancers are not known.

In the present study, we screened a panel of sarcoma cell lines and patient-derived tissue and show that TBX3 is highly expressed in sarcomas representative of diverse histological subtypes and that, similar to its role in carcinomas, it promotes migration of chondrosarcoma, liposarcoma and rhabdomyosarcoma cells. Interestingly, we found TBX3 to inhibit migration of fibrosarcoma cells, suggesting that it may function to either promote or inhibit tumorigenesis depending on the cellular context. We further explore this possibility by establishing and characterizing cell culture models in which TBX3 is either knocked down or overexpressed in chondrosarcoma and fibrosarcoma cell lines. Similar to what has been described for TBX3 in carcinomas we show that it directly contributes to the oncogenic phenotype of chondrosarcoma cells. Importantly, we provide evidence for a novel tumour suppressor role for TBX3 in fibrosarcomas where it inhibits cell proliferation, migration and tumour-forming ability. Taken together, this study shows for the first time that TBX3 is overexpressed in several sarcoma subtypes and that it functions as either an oncoprotein or a tumour suppressor depending on the cellular context. Our findings suggest that TBX3 may be a candidate diagnostic marker and a common therapeutic target for a diverse range of sarcoma subtypes.

## Results

### TBX3 is overexpressed in soft tissue and bone sarcomas

**Figure 1:**
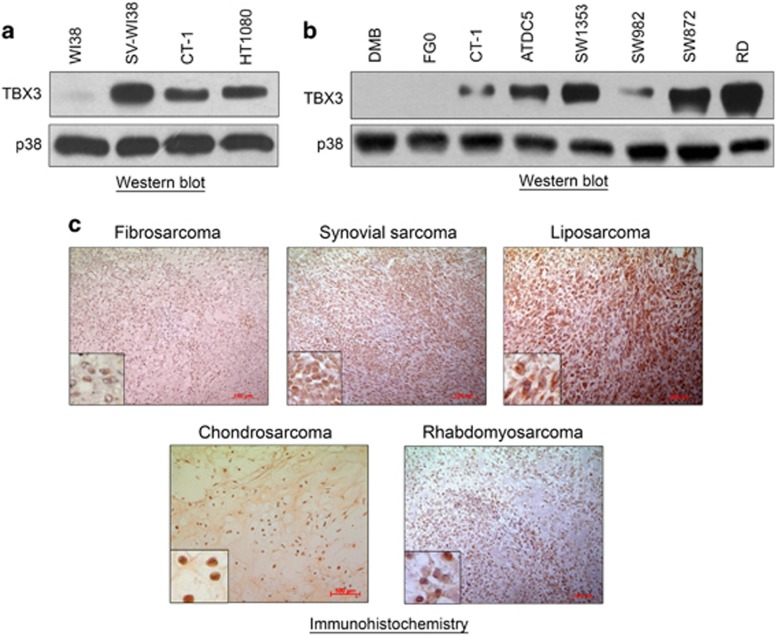
TBX3 is overexpressed in soft tissue and bone sarcomas. Protein from (**a**) the WI38 normal human fibroblast, transformed SV-WI38 and CT-1 fibroblast, and HT1080 fibrosarcoma cell lines and (**b**) the DMB and FGO normal human fibroblast, CT-1 transformed fibroblast, ATDC5 and SW1353 chondrosarcoma, SW982 synovial sarcoma, SW872 liposarcoma and RD rhabdomyosarcoma cell lines were screened for TBX3 expression using western blotting with an antibody specific to TBX3. p38 was used as a loading control. (**c**) Archival patient-derived fibrosarcoma (*N*=4), synovial sarcoma (*N*=2), liposarcoma (*N*=3), chondrosarcoma (*N*=1) and rhabdomyosarcoma (*N*=3) tissue sections were immunohistochemically stained using an antibody specific to TBX3. Representative images are shown (scale bars, 100 μm; insets are magnified images from selected areas).

To begin to explore the status of TBX3 in sarcomas we firstly analysed TBX3 expression in a panel of normal and transformed fibroblast cell lines by western blotting. Compared with the normal WI38 fibroblast cells, TBX3 was upregulated in transformed (CT-1 and SV-WI38) fibroblasts as well as the naturally occurring HT1080 human fibrosarcoma cells (Figure 1a). We next determined if this overexpression of TBX3 may be a feature of sarcomas and to this end we screened a panel of soft tissue and bone sarcomas for TBX3 protein. Indeed, compared with the normal human skin fibroblast cell lines, FG0 and DMB, TBX3 was highly expressed in chondrosarcoma (ATDC5 and SW1353), synovial sarcoma (SW982), liposarcoma (SW872) and embryonal rhabdomyosarcoma (RD) cell lines (Figure 1b). Furthermore, immunohistochemical analyses revealed that TBX3 was expressed in patient-derived fibrosarcoma, synovial sarcoma, liposarcoma, chondrosarcoma and rhabdomyosarcoma tissue sections (Figure 1c).

### TBX3 impacts on sarcoma cell migration

**Figure 2:**
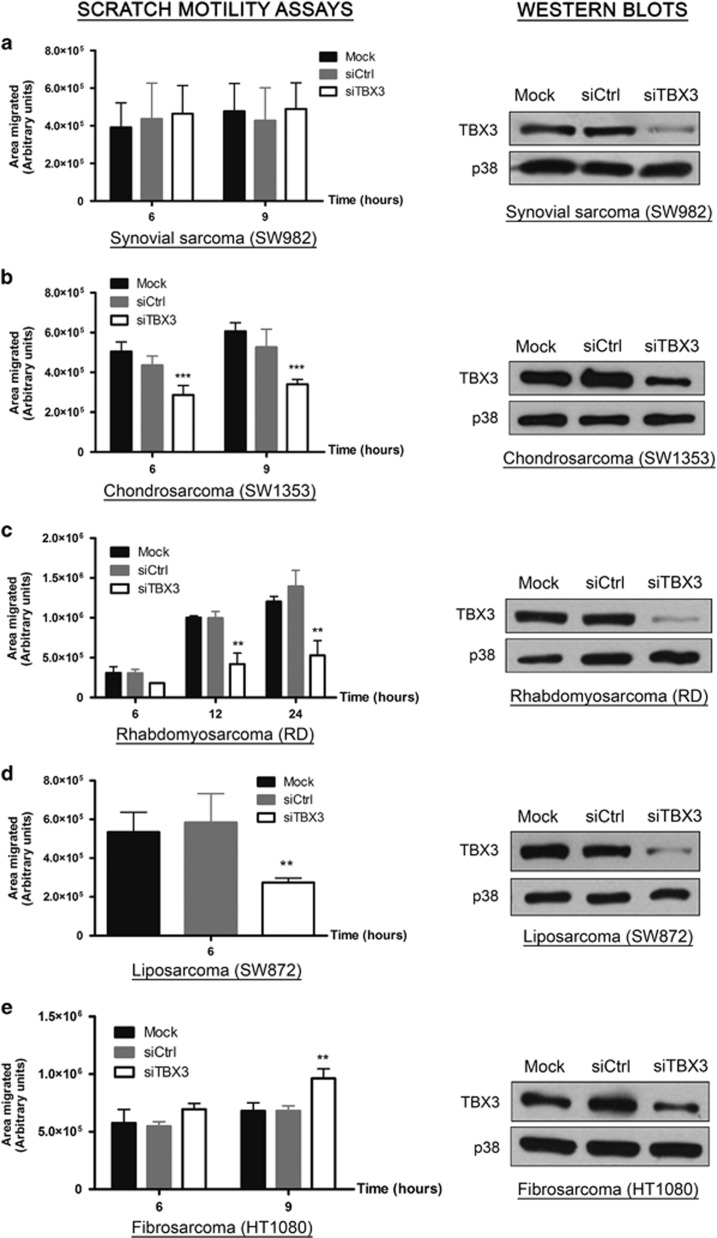
TBX3 impacts on sarcoma cell migration. Left panels: (**a**) SW982 synovial sarcoma, (**b**) SW1353 chondrosarcoma, (**c**) RD rhabdomyosarcoma, (**d**) SW872 liposarcoma and (**e**) HT1080 fibrosarcoma cell lines were transfected with transfection reagent only (mock), control siRNA or siTBX3 and 48 h later scratch motility assays were performed. Data are the mean±s.d. of three independent experiments, **P<0.01; ***P<0.001. Right panels (**a**–**e**): Western blot analyses were performed to assess TBX3 knockdown using an antibody specific to TBX3 and p38 was used as a loading control.

TBX3 has a well-established role in promoting migration of several carcinomas where it is overexpressed.^15, 20, 21, 22, 23, 24^ In light of our results showing that TBX3 is upregulated in sarcomas, we therefore next investigated whether it also impacts on the migration of sarcoma cells. To this end, we transiently knocked down TBX3 using siRNA (small interfering RNA) in five histologically diverse sarcoma subtypes and performed scratch motility assays. While TBX3 depletion had no effect on the migratory ability of synovial sarcoma (SW982) cells (Figure 2a), it led to a significant reduction in the migration of chondrosarcoma (SW1353) (Figure 2b), rhabdomyosarcoma (RD) (Figure 2c) and liposarcoma (SW872) (Figure 2d) cells. Quite unexpectedly, knocking down TBX3 resulted in increased migration of fibrosarcoma (HT1080) cells (Figure 2e).

### Establishment of chondrosarcoma and fibrosarcoma cell lines in which TBX3 was either stably knocked down or overexpressed

**Figure 3:**
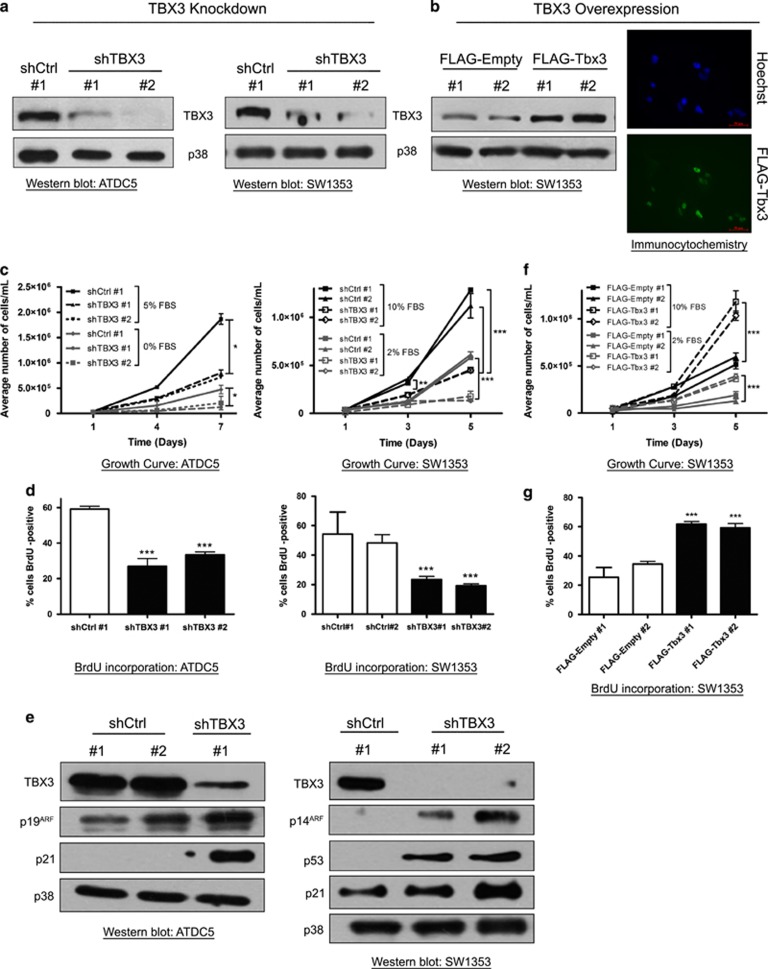
TBX3 promotes proliferation of chondrosarcoma cells by repressing key cell cycle regulators. (**a**, **b**) Protein extracts from indicated cell lines were analysed by western blotting using an antibody specific to TBX3 and p38 was used as a loading control. (**b**) Right panel, immunocytochemistry with an antibody specific to FLAG shows TBX3 overexpression in SW1353 FLAG-Tbx3 cells. Hoechst was used to stain the nuclei. Representative images are shown (scale bars, 50 μm). (**c**, **f**) Growth curve analyses of (**c**) shCtrl and shTBX3 cells and (**f**) FLAG-empty and FLAG-Tbx3 cells. (**d**, **g**) Cells were pulsed with BrdU and processed for immunocytochemistry using an antibody specific to BrdU and visualized by fluorescence microscopy. Bar graphs show the average percentage of BrdU-positive cells in 20 fields of view. (**e**) Western blotting with antibodies specific to TBX3, p14^ARF^, p53 and p21. p38 was used as a loading control. (**c**, **d**, **f**, **g**) Data are the mean±s.d. of three independent experiments, *P<0.05; ***P<0.001.

The above data gave an initial indication that TBX3 may have different oncogenic roles in sarcomas and we therefore further investigated this in fibrosarcoma and chondrosarcoma cell lines in which TBX3 was either knocked down or overexpressed. To knock down TBX3, cells were stably transfected with a pSuper.neo/GFP expression vector containing a short-hairpin (sh) RNA sequence targeting TBX3 (shTBX3) or a nonspecific control sequence (shCtrl). A number of G418-resistant clones were isolated and TBX3 knockdown was confirmed by western blotting and the clones further characterized are shown in Figures 3a and 6a. Stable cell lines in which TBX3 was ectopically overexpressed were generated by transfecting cells with a FLAG-tagged pCMV-Tbx3 or a control pCMV-Empty vector and western blotting and immunocytochemistry show the G418-resistant clones overexpressing TBX3 that were used for further analyses (Figures 3b and 6b).

### TBX3 promotes proliferation of chondrosarcoma cells by repressing key cell cycle regulators

To determine the effect of TBX3 on chondrosarcoma cell proliferation, growth curve analyses and 5-bromo-2-deoxyuridine (BrdU) incorporation assays were performed and results showed that compared with their control cells, ATDC5 and SW1353 shTBX3 chondrosarcoma cells exhibited a significantly slower growth rate under normal and reduced serum conditions (Figures 3c and d). Consistent with this finding, levels of the previously identified TBX3 targets, p14/p19^ARF^ and p21, increased in both shTBX3 chondrosarcoma cell lines (Figure 3e), which suggest that TBX3 promotes proliferation of chondrosarcoma cells by, in part, repressing key cell cycle regulators.^18, 19^ It is worth noting that knockdown of TBX3 in the SW1353, but not the ATDC5 (data not shown), cells also resulted in increased p53 protein levels, which suggest that the proproliferative ability of TBX3 in chondrosarcoma cells may be both p53 dependent and independent.

In support of the above data, when TBX3 was ectopically overexpressed in chondrosarcoma cells the proliferative ability of the cells increased (Figures 3f and g) which correlated with, as expected, undetectable levels of p53, p21 and p14^ARF^ (data not shown).

### TBX3 is required for anchorage-independent growth, migration and *in vivo* tumour-forming ability of chondrosarcoma cells

**Figure 4:**
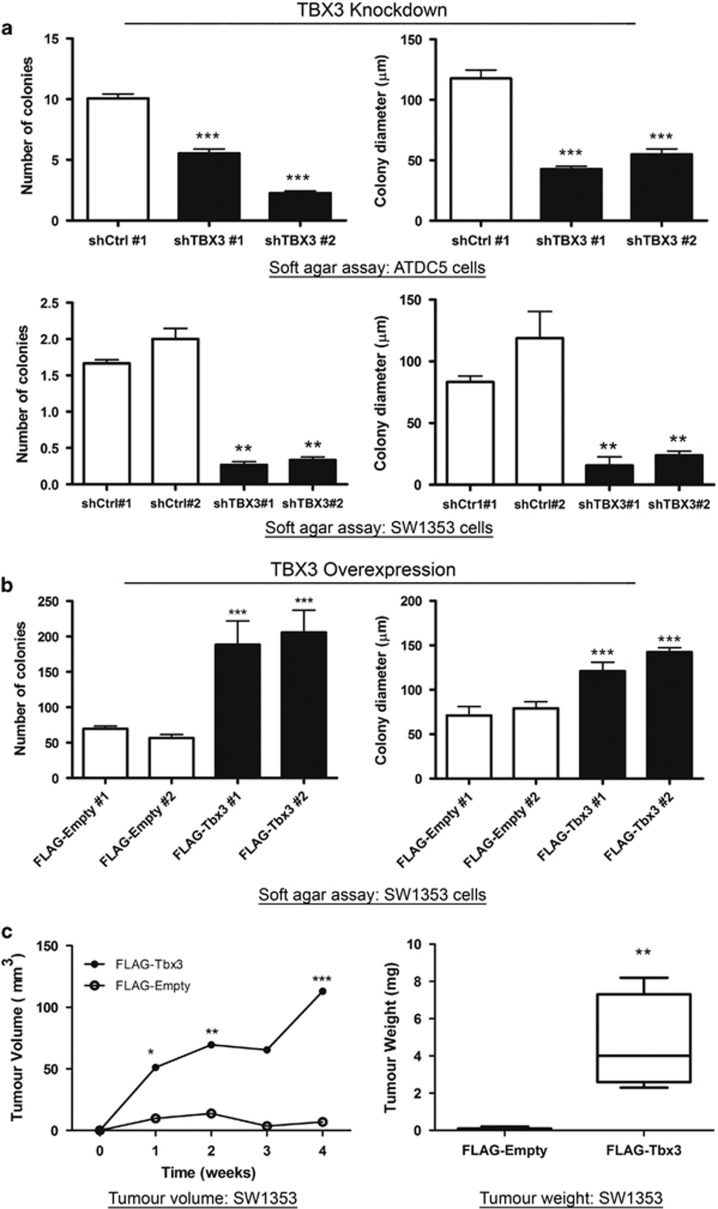
TBX3 promotes anchorage-independent growth and *in vivo* tumour-forming ability of chondrosarcoma cells. Cell growth in soft agar of (**a**) ATDC5 (top) and SW1353 (bottom) shCtrl and shTBX3 cells and (**b**) SW1353 FLAG-Empty and FLAGTbx3 cells was assessed by staining viable colonies with *p*-iodinitrotetrazolium chloride. Quantitative analyses of number of colonies and colony diameter were calculated from 20 fields of view. (**a**, **b**) Data are the mean±s.d. of three independent experiments, *P<0.05; **P<0.01; ***P<0.001. (**c**) Left panel: SW1353 FLAG-Empty and FLAG-Tbx3 cells were subcutaneously injected into the flanks of NSG immunocompromised mice (*N*=5 each). *In situ* tumour volume (mm^3^) was measured using callipers. Right panel: Following euthanasia, tumours were excised and weighed (grams). Data represent mean±s.d. (*N*=5 each), *P<0.05; **P<0.01; ***P<0.001.

We next determined the impact of TBX3 on anchorage-independent growth of chondrosarcoma cells using soft agar assays. Our results show that in the absence of a substrate shTBX3 cells had reduced proliferative ability and formed significantly fewer and smaller colonies (Figure 4a). On the other hand, compared with their control cells, the SW1353 cells that ectopically overexpress TBX3 (SW1353 FLAG-Tbx3) formed more and larger colonies (Figure 4b). Consistent with this ability of TBX3 to promote anchorage-independent growth *in vitro*, when NOD scid gamma (NSG) mice were injected subcutaneously with SW1353 FLAG-Tbx3 cells or FLAG-Empty cells, the tumour volume and weight for cells overexpressing TBX3 were significantly greater (Figure 4c)

**Figure 5:**
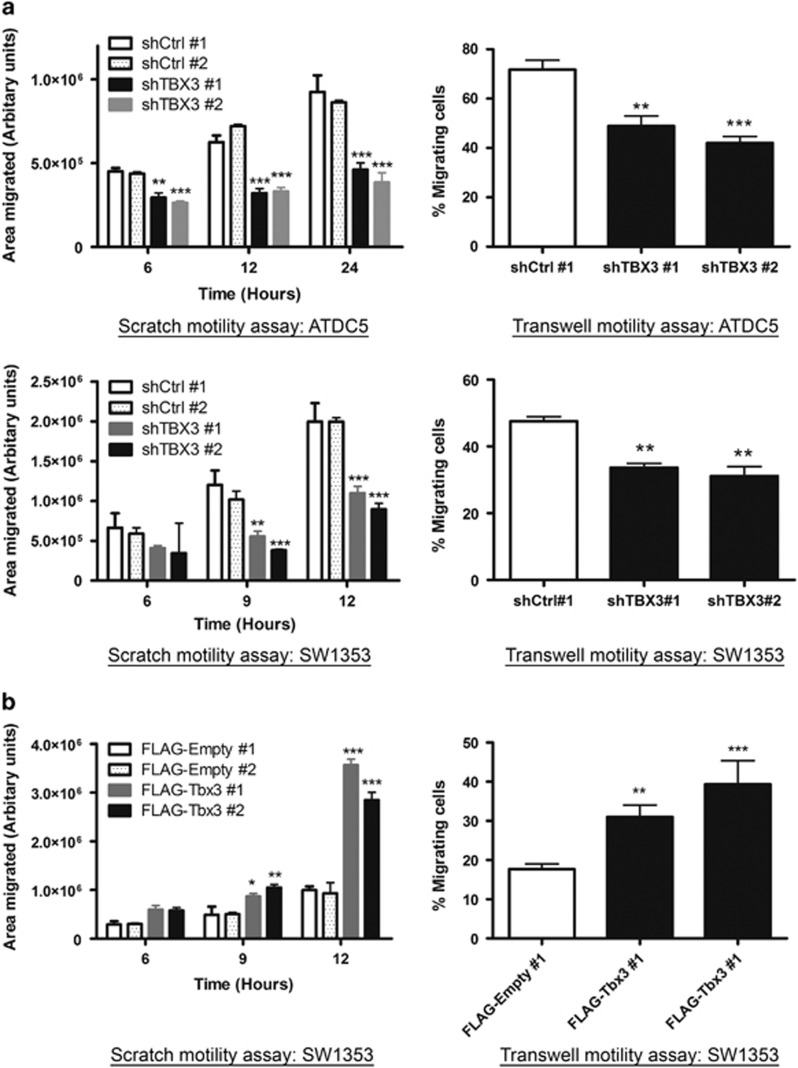
TBX3 promotes migration of chondrosarcoma cells. Scratch (left panels) and transwell (right panels) motility assays were performed to measure the migration of (**a**) shCtrl and shTBX3 ATDC5 cells (top panel) and shCtrl and shTBX3 SW1353 cells (lower panel) and (**b**) SW1353 FLAG-Empty and FLAG-Tbx3 cells. (**a**, **b**) Data are the mean±s.d. of three independent experiments, **P<0.01; ***P<0.001.

Results shown in Figure 2 suggested that TBX3 promotes migration in chondrosarcoma cells. To confirm this in our chondrosarcoma cell lines in which TBX3 was either stably knocked down or overexpressed, we performed scratch and transwell motility assays. As expected, whereas depleting TBX3 inhibited the migration of chondrosarcoma cells (Figure 5a), ectopic overexpression of TBX3 promoted their migration (Figure 5b). Together these results provide compelling evidence to support a role for TBX3 as an oncogene in chondrosarcomas.

### TBX3 exhibits tumour suppressor activity in fibrosarcoma cells *in vitro*

**Figure 6:**
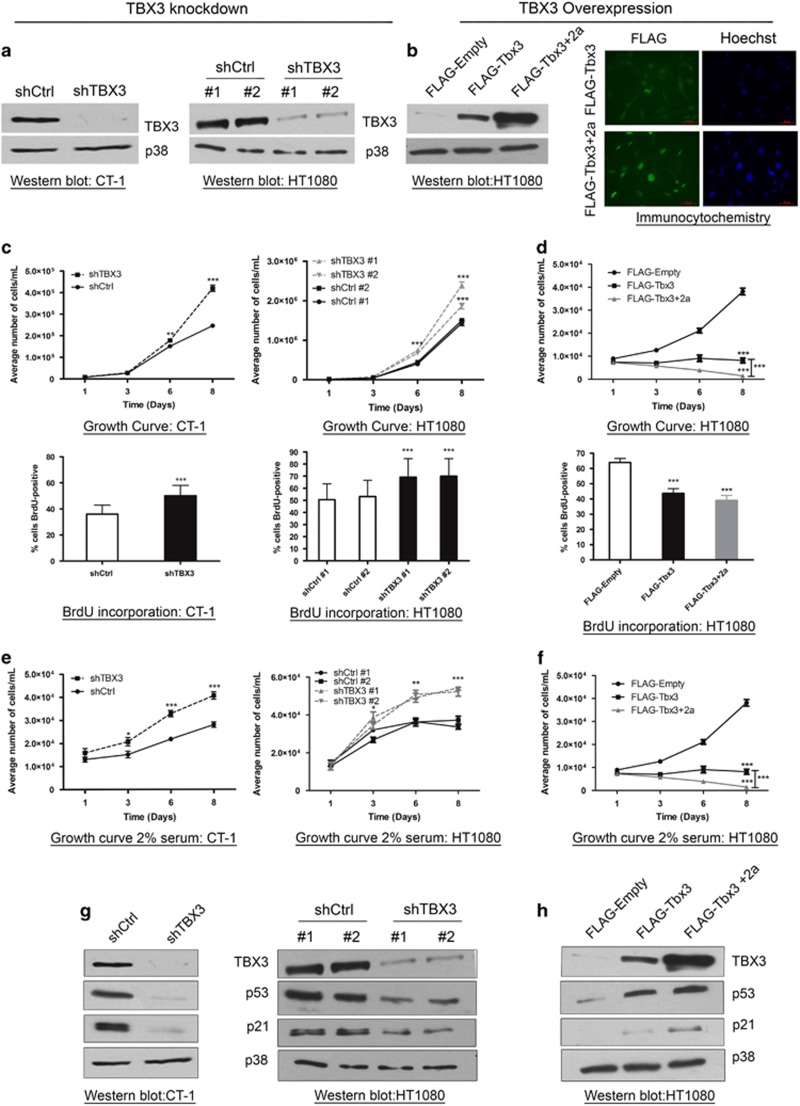
TBX3 represses proliferation of fibrosarcoma cells by activating key cell cycle regulators. (**a**, **b**) Protein extracts from indicated cell lines were analysed by western blotting using an antibody specific to TBX3 and p38 was used as a loading control. (**b**) Right panel, immunocytochemistry with an antibody specific to FLAG shows TBX3 overexpression in HT1080 FLAG-Tbx3 cells and FLAG-Tbx3+2a. Hoechst was used to stain the nuclei. Representative images are shown (scale bars, 50 μm). (**c**–**f**) Growth curve analyses and BrdU incorporation assays for (**c**, **e**) shCtrl and shTBX3 and (**d**, **f**) FLAG-empty and FLAG-Tbx3 cells. For the BrdU incorporation assays cells were pulsed with BrdU and processed for immunocytochemistry using an antibody specific to BrdU and visualized by fluorescence microscopy. Bar graphs show the average percentage of BrdU-positive cells in 20 fields of view. (**c**–**f**) Data are the mean±s.d. of three independent experiments, *P<0.05; **P<0.01; ***P<0.001. (**g**, **h**) Western blotting with antibodies specific to TBX3, p53 and p21. p38 was used as a loading control.

To further explore the putative tumour suppressor activity of TBX3 in fibrosarcomas, we characterized the impact of stably knocking down TBX3 in CT-1 and HT1080 fibroblasts (Figure 6a) or overexpressing the Tbx3 and Tbx3+2a isoforms in the HT1080 cells (Figure 6b) on key features of the cancer phenotype. The two Tbx3 isoforms were included because they may have opposite effects on oncogenesis, which may account for the unexpected tumour suppressor function observed in Figure 2. Growth curve analyses and BrdU incorporation assays show that compared with control cells, shTBX3 cells had significantly enhanced proliferative ability while both FLAG-Tbx3 and FLAG-Tbx3+2a cells grew significantly more slowly than their control cells (Figures 6c and d). A similar trend was seen when cells were cultured under reduced serum conditions (Figures 6e and f). The negative impact of TBX3 on the proliferative ability of fibroblasts correlated with levels of the key cell cycle regulators p53 and p21 (Figures 6g and h). Taken together these results suggest that TBX3 may inhibit fibroblast cell proliferation by activating p53 and its downstream target p21 and that the TBX3 and TBX3+2a isoforms are functionally similar in this context.

**Figure 7:**
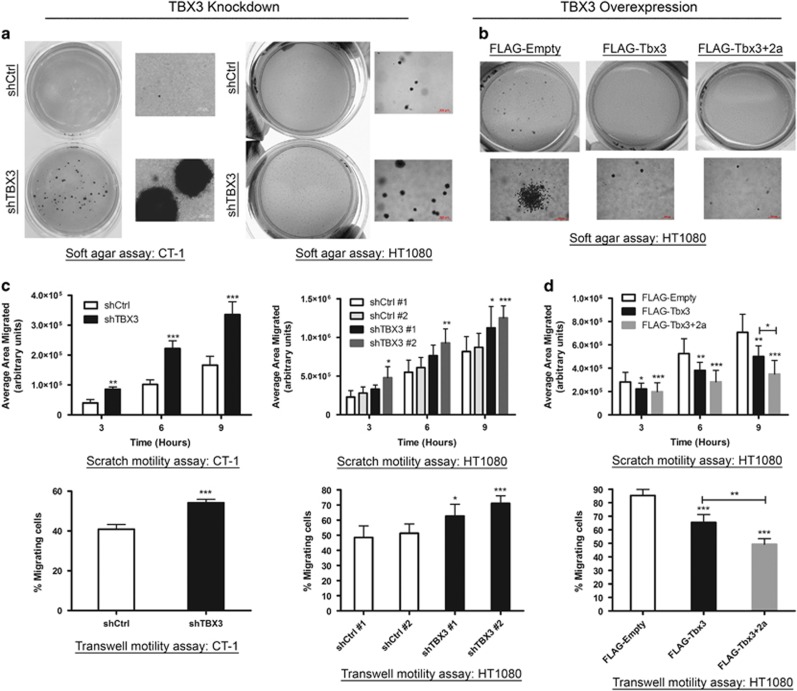
TBX3 represses anchorage-independent growth and migration of fibrosarcoma cells. (**a**) CT-1 (left) and HT1080 (right panel) shCtrl and shTBX3 cells and (**b**) HT1080 FLAG-Empty, FLAG-Tbx3 and FLAG-Tbx3+2a cells were suspended in soft agar-medium slurry and allowed to proliferate for 21–35 days. Whole dishes were stained with *p*-iodinitrotetrazolium chloride to indicate viable populations and images of colonies were taken at × 10 magnification. (**c**) Migration of CT-1 (left) and HT1080 (right) shCtrl and shTBX3 cells and (**d**) HT1080 FLAG-empty, FLAG-Tbx3 and FLAG-Tbx3+2a cells was analysed using scratch (top) and transwell motility assays (bottom). For the transwell assay the results show the percentage of cells that migrated through the transwell insert. (**c**, **d**) Data are the mean±s.d. of three independent experiments, *P<0.05; **P<0.01; ***P<0.001.

Consistent with it having antitumour activity in fibroblasts, depleting TBX3 led to an increase in their anchorage-independent cell proliferation in soft agar assays (Figure 7a) while overexpressing either TBX3 isoform had the opposite effect (Figure 7b). Similarly, while knocking down TBX3 enhanced the migratory ability of both CT-1 and HT1080 cells in motility assays (Figure 7c), overexpressing either isoform reduced this ability (Figure 7d). It is worth noting that compared with Tbx3 the activity of the Tbx3+2a isoform appears more pronounced. We believe that this is due to the different levels of overexpression achieved for the two isoforms, that is, Tbx3+2a expressed at much higher levels than Tbx3 (see Figures 6b and h). Together these results confirm that TBX3 and TBX3+2a have tumour suppressor activity in fibroblasts *in vitro*.

### TBX3 exhibits tumour suppressor activity in fibrosarcoma cells *in vivo*

**Figure 8:**
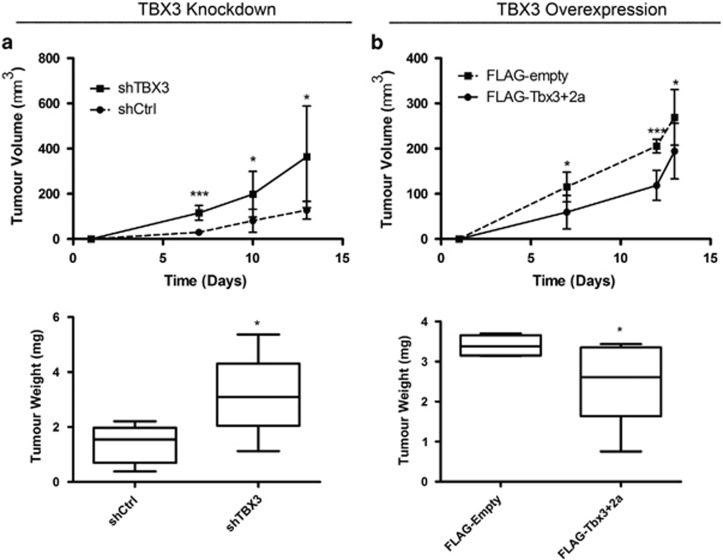
TBX3 represses *in vivo* tumour-forming ability of fibrosarcoma cells. (**a**) HT1080 shCtrl and shTBX3 cells and (**b**) HT1080 FLAG-empty and FLAG-Tbx3+2a cells were subcutaneously injected into the flanks of MF-1 nude mice. Top: *In situ* tumour volume (mm^3^) was measured using callipers. Bottom: Following euthanasia, tumours were excised and weighed (g). (**a**, **b**) Data represent mean±s.d. (*N*=5 each), *P<0.05; ***P<0.001.

To confirm that TBX3 functions as a tumour suppressor in fibrosarcomas *in vivo*, HT1080-shTBX3, HT1080-FLAG-Tbx3+2a and their control cells were injected subcutaneously into the right flank of nude mice and tumour growth was monitored *in situ* over 2 weeks. While both the HT1080 shCtrl and HT1080-shTBX3 cells were able to form tumours, the volume and average mass of those produced by the shTBX3 cells were significantly greater (Figure 8a). Furthermore, the overexpression of Tbx3+2a was able to significantly reduce the tumour volume and weight (Figure 8b). Histopathological analyses revealed the tumours to be spindle cell masses consistent with fibrosarcomas and sections stained positive for the mesenchymal marker, vimentin (data not shown). Taken together, data from our chondrosarcoma and fibrosarcoma cell culture models provide compelling evidence for a novel tumour suppressor role for TBX3 in fibroblasts and suggest that TBX3 may play opposite roles in the development of sarcomas.

## Discussion

There is a significant body of evidence implicating TBX3 as an oncoprotein in several carcinomas; however, nothing is known about its status and role in sarcomas.^12, 13, 24, 25, 26, 27, 28, 29^ Here we show that TBX3 is highly expressed in a panel of soft tissue and bone sarcoma cell lines and patient-derived sarcoma tissue. Significantly, chondrosarcoma and fibrosarcoma cell culture models in which TBX3 was either depleted or overexpressed revealed that while TBX3 contributes to cell proliferation, migration and tumour formation in chondrosarcoma cells, it has an inhibitory effect on these processes in fibrosarcomas. A positive effect of TBX3 on cell migration was also observed in liposarcoma and rhabdomyosarcoma cells. Together these findings provide evidence that the overexpression of TBX3 may be a feature of a wide range of sarcoma subtypes and that TBX3 impacts directly on their development as either an oncogene or a tumour suppressor.

Sarcomas represent a diverse cluster of malignancies with vastly different biology and clinical behaviour and this presents a serious obstacle to early and reliable diagnosis as well as therapy.^30, 31, 32^ In addition, many sarcoma subtypes are associated with poor prognosis due to resistance to conventional therapies such as surgery, chemotherapy and radiation, and hence a major goal in sarcoma research has been to develop molecular targetted therapies.^30, 31, 32^ Our observations that TBX3 expression is elevated in sarcoma cell lines and patient-derived tissue sections representative of both simple (synovial sarcoma) and complex (fibrosarcoma, chondrosarcoma, liposarcoma and embryonal rhabdomyosarcoma) karyotypes suggest that TBX3 is a common feature in multiple signalling networks involved in sarcomagenesis.^32^ This raises the possibility that TBX3 may represent a promising diagnostic marker for a diverse range of sarcoma subtypes. Moreover, early and accurate diagnosis of sarcomas is often masked by the high occurrence of benign soft tissue masses that largely outnumber malignant sarcomas.^33, 34^ Our observation that TBX3 is expressed in tumour cells and tissues indicates that TBX3 expression may be useful to differentiate sarcomas from benign soft tissue masses which will assist with appropriate treatment planning. However, the sample size of our study was small due to the rarity of these tumours and future studies will be necessary to confirm our observations in many more patient samples. Furthermore, our data showing that depleting TBX3 inhibits the cancer phenotype of several sarcoma subtypes suggests that it may also represent a common target to treat diverse sarcomas.

We and others have previously shown that TBX3 promotes migration of breast and bladder carcinomas and melanoma cells by a process involving its ability to repress the cell adhesion molecule, E-cadherin.^20, 23, 24, 35^ Here we show that TBX3 similarly impacts on migration of chondrosarcoma, liposarcoma and rhabdomyosarcoma cells, but they did not however express detectable levels of E-cadherin (data not shown). This would be consistent with numerous other studies that have shown that only a fraction of sarcomas express E-cadherin and indeed 90% of epithelioid sarcoma cases were reported to be E-cadherin negative.^36^ The molecular mechanism(s) underlying the promigratory role of TBX3 in carcinomas and sarcomas therefore appears to be different and future studies identifying TBX3 target genes as well as signalling pathways that upregulate TBX3 in sarcomas would likely shed light on this. Of interest would be the Wnt/β-catenin and PI3K/Akt signalling pathways because they promote metastasis of a number of sarcoma subtypes.^31, 37, 38^ For example, high levels of β-catenin and aberrant activation of Wnt signalling are frequently observed in sarcomas and this pathway promotes sarcoma metastasis by modulating the Wnt target genes, MMP-9 and c-Myc.^39^ Furthermore, constitutive activation of AKT, in part due to the repression of PTEN, is observed in 55% of soft tissue sarcoma cases^31, 40^ and targeting AKT was shown to reduce pulmonary metastasis in osteosarcoma bearing mice.^41^ Interestingly, TBX3 is positively regulated by AKT, Wnt/β-catenin and c-Myc in melanoma, liver cancer and chondrosarcomas, respectively, and it directly represses PTEN to promote head and neck carcinomas.^13, 42, 43^ It will thus be worth investigating whether these signalling networks regulate TBX3-induced sarcoma cell migration.

Unexpectedly, our study also revealed a novel role for TBX3 as a tumour suppressor in fibrosarcomas. Indeed, whereas knocking down TBX3 in transformed fibroblasts resulted in a more aggressive cancer phenotype, ectopic overexpression of Tbx3, or its splice variant Tbx3+2a, was sufficient to inhibit key features of the cancer phenotype in the aggressive HT1080 cell line. While this is the first study to provide a full characterization of a tumour suppressor function for TBX3, there are a few high-throughput studies that have hinted at this possibility. Using microarray analyses, Lyng *et al.*^29^ showed that TBX3 expression was downregulated in cervical and uterine cancer samples and that this strongly correlated with lymph node metastasis and reduced progression-free survival. In addition, the silencing of TBX3 by methylation has been associated with progression to muscle-invasive bladder tumours and with more aggressive prostate tumours and this correlated with significantly lowered survival rate.^44, 45, 46, 47^ The apparent paradoxical ability of TBX3 to either promote or inhibit tumorigenesis has also been reported for other developmental transcription factors with prime examples including FOXO3,^48, 49^ TGFβ,^50^ Sox4^51^ and other T-box factors. For example, while the overexpression of Brachyury contributes to a number of tumour types through its ability to promote epithelial–mesenchymal transition,^52, 53^ a high-throughput study has revealed that Brachyury is epigenetically silenced in lung cancer and may be a tumour suppressor.^54^ TBX5 has also been shown to induce apoptosis and inhibit tumour formation in osteosarcoma, lung and colon cancer,^55, 56^ but also to interact with YAP1 and β-catenin to activate the expression of a number of antiapoptotic genes in β-catenin-driven cancers.^57^ Taken together, our findings reveal that, depending on cellular context, TBX3 plays opposite roles in cancer and it will be important to elucidate the mechanism(s) that enables it to switch between these functions. We speculate that it involves protein co-factors and studies are therefore underway to identify TBX3 interacting partners in chondrosarcomas and fibrosarcomas.

In summary, results from this study contribute significantly to an understanding of the role of TBX3 in cancer biology and provide new evidence that TBX3 also impacts on sarcomagenesis.

## Materials and methods

### Cell culture

Human embryonic lung fibroblast WI38 cells (ATCC CCL-75), γ-radiation transformed WI38 fibroblast cells (CT-1),^58^ SV40 transformed WI38 cells (SV-WI38),^59^ HT1080 human fibrosarcoma cells (ATCC CCL-120), FG0 and DMB human skin fibroblasts,^60^ SW1353 human chondrosarcoma cells (ATCC HTB-94), SW982 human synovial sarcoma cells (ATCC HTB-93), SW872 human liposarcoma (ATCC HTB-92) and RD human embryonal rhabdomyosarcoma cell (ATCC CCL-136) were cultured in Dulbecco's modified Eagle's medium (DMEM) (Sigma Aldrich, St Louis, MO, USA), supplemented with 10% heat-inactivated foetal bovine serum (FBS), 100 U/ml penicillin and 100 μg/ml streptomycin. ATDC5 mouse chondrosarcoma cells were maintained in DMEM:nutrient mixture F-12 (DMEM/F-12; 1:1; Sigma Aldrich), supplemented with 5% FBS, 100 U/ml penicillin, 100 μg/ml streptomycin, 10 μg/ml human transferrin (Sigma Aldrich) and 3 × 10^−8^ m sodium selenite (Sigma Aldrich). All cells were maintained as previously described.^20^

### Immunohistochemistry

Paraffin-embedded tissue sections (*N*=13) were obtained from the Division of Anatomical Pathology, University of Cape Town and this study was approved by and performed in accordance with the University of Cape Town Human Research Ethics Committee. Paraffin-embedded tumour tissues from surgical specimens were cut in 5-μm-thick sections. Antigen retrieval was performed with citric acid buffer at pH 6 for 90 s using a pressure cooker and cooled for an additional 30 min. Tissue sections were blocked with 5% goat serum (X090710; Dako, Glostrup, Denmark) in phosphate-buffered saline and incubated with rabbit polyclonal anti-TBX3 (1:25; Zymed, Invitrogen, Carlsbad, CA, USA) overnight. Secondary antibody (K400211; Dako) and DAB chromogen (K346711; Dako) were applied according to the manufacturer's instructions. Slides were counrtstained with hematoxylin, the nuclei stained with Scott's solution and mounted using Entellan (107960; Merck, Darmstadt, Germany).

### Small interferring RNA

Transient suppression of TBX3 cellular expression was achieved using 50 nm siRNA specifically designed to target TBX3 mRNA. The cells were transfected with siTBX3 (SI00083503; Qiagen, Valencia, CA, USA) or a control (non-silencing) siRNA (1027310; Qiagen) using HiPerFect (Qiagen) according to the manufacturer's instructions.

### Generation of stable cell lines

For the generation of stable TBX3 knockdown lines, CT-1, HT1080, SW1353 and ATDC5 cells were stably transfected with a pSuper.neo/GFP expression vector containing a sequence targeted to TBX3 or a nonspecific control, as previously described.^20^ Stable transfectants were selected in growth medium containing 400 μg/ml G418 (Promega, Madison, WI, USA) (ATDC5 and CT-1 cells) or 800 μg/ml G418 (HT1080 and SW1353 cells). Effective knockdown of TBX3 was assessed by western blot analysis. TBX3 overexpressing cell lines were generated by stably transfecting HT1080 and SW1353 cells with a FLAG-tagged pCMV-empty vector or pCMV constructs expressing the mouse Tbx3 (pCMV-Tbx3) or mouse Tbx3+2a (pCMV-Tbx3+2a) (kindly provided by Professor Colin Goding at the Ludwig Institute of Cancer Research, Oxford, London, UK) using FuGENEHD (Roche Molecular Biochemicals, Mannheim, Germany) according to the manufacturer's instructions. Stable transfectants were selected for with 800 μg/ml G418 antibiotic (Promega). Effective overexpression of Tbx3 and Tbx3+2a was assessed by western blot analysis.

### Western blot analysis

Cells were harvested and protein prepared as described previously.^61^ Primary antibodies used were as follows: rabbit polyclonal anti-TBX3 (42-4800; Zymed, Invitrogen), rabbit polyclonal anti-p38 (M0800) and mouse monoclonal anti-FLAG M2 (F1804; Sigma, St Louis, MO, USA), rabbit polyclonal p19^ARF^ (sc-1063), mouse polyclonal anti-p53 (sc-6243), rabbit polyclonal anti-p21 (C-19, Santa Cruz Biotechnology, Santa Cruz, CA, USA).

### BrdU incorporation assay

BrdU incorporation assays were performed as described previously^61^ using 10 μm BrdU and a mouse monoclonal anti-BrdU antibody (6 μg/ml; Roche), followed by a secondary IgG coupled to Alexa-488 (1:1000; Molecular Probes, Carlsbad, CA, USA). Slides were mounted with Mowial mounting medium and visualized by fluorescence microscopy using an Axiovert fluorescent microscope (Zeiss, Oberkochen, Germany). Data were obtained from three independent experiments.

### Proliferation assays

Short-term growth of the TBX3 knockdown and overexpression lines was performed in DMEM supplemented with 10% or 2% FBS (CT-1, HT1080 and SW1353 cells) or 5% or 0% FBS (ATDC5 cells). Cells were plated in triplicate in 12-well plates as follows: 1 × 10^4^ per well for CT-1 cells and 0.5 × 10^4^ cells per well for HT1080, SW1353 and ATDC5 cell lines. Growth curves were performed over an 8-day period, as previously described.^62^ Data were obtained from three independent experiments.

### Anchorage-independent assay

Soft agar assays were performed as described previously.^61^ Dishes (35 mm) were layered first with 0.5% agar in cell culture medium followed by 0.35% agar in cell culture medium containing 5000 cells. Colonies were stained with *p*-iodonitrotetrazolium chloride (1 mg/ml), incubated overnight at 37 °C and photographed. Data were obtained from three independent experiments.

### Cell migration assays

Cell migration was measured using a two-dimensional *in vitro* scratch motility assay as previously described.^20^ The wound areas were measured over time and calculated using ImageJ software (National Institutes of Health, Bethesda, MD, USA). For the transwell assay, transwell plates with an 8- μm pore size were used (ThinCert cell culture inserts; Kremsmünster, Greiner, Austria). Cells were seeded at 1 × 10^5^ cells in the top chamber, in DMEM supplemented with 2% FBS and incubated for 24 h at 37 °C. The bottom chamber contained DMEM supplemented with 10% FBS. Twenty-four hours later, cells were fixed in 100% methanol and cotton swabs were used to remove cells in the upper surface of the transwells. Migrated cells attached to the undersurface of the transwell were stained with crystal violet solution and then air-dried. Crystal violet-stained cells were solubilized in 50% acetic acid and quantified using a microplate reader at 595 nm. Data were obtained from three independent experiments.

### Xenograft mouse models

All protocols employed in this study were approved by and performed in accordance with the University of Cape Town Animal Research Ethics Committee guidelines for care and use of laboratory animals. Unblinded tumorigenicity experiments were performed by subcutaneously injecting 1 × 10^7^ SW1353 FLAG-Empty or FLAG-Tbx3 cells in 100;μl phosphate-buffered saline into the right flanks of randomly selected 4- to 6-week-old NOD/Lt-scid/IL2Rγ^null^ (NSG) mice (*N*=5 per group) (The Jackson Laboratory, Bar Harbor, ME, USA). For HT1080 cells, 5 × 10^6^ shCtrl or shTBX3 cells or 4 × 10^6^ FLAG-Empty or FLAG-Tbx3+2a cells were injected into the right flanks of randomly selected 6-week-old MF-1 nude mice (*N*=5 per group). Tumour growth was measured using the formula (volume mm^3^=(length) × (width^2^) x 0.5). Once tumour volume had reached a length of 12mm, mice were killed and organs, including tumours, were removed for histopathological analyses (IDEXX Laboratories (Pty) Ltd, Cape Town, South Africa). Power analysis to determine sample size was performed using PS (Power and Sample size) freeware.

### Immunocytochemistry

Cells were grown on glass coverslips and fixed with flash treatment of ice-cold methanol before permeabilization with 0.25% Triton X-100 in phosphate-buffered saline for 5 min at room temperature. Slides were incubated with mouse monoclonal anti-FLAG M2 antibody (1:500; F1804; Sigma) for 2 h at 37 °C, followed by incubation with the appropriate secondary antibody coupled to Alexa-488 (1:1000; Jackson ImmunoResearch Laboratories Inc., West Grove, PA, USA). All cells were co-stained with Hoechst (33342; Invitrogen). Cells were mounted using Mowiol mounting medium and examined by fluorescence microscopy using an Axiovert fluorescent microscope (Zeiss).

### Statistical analysis

Statistical significance was determined using Student's t-test (Excel, Microsoft, Redmond, WA, USA) or two-way ANOVA (Graphpad Prism 4, San Diego, CA, USA). Significance was accepted at *P*<0.05. Power analysis to determine sample size for xenograft studies was performed using PS (Power and Sample size) freeware.
